# The Impact of Process Quality in Early Childhood Education and Care on Socio-Emotional Development: A Meta-Analysis of Longitudinal Studies

**DOI:** 10.3390/ijerph22050775

**Published:** 2025-05-14

**Authors:** Rosanne M. V. Sluiter, Ruben G. Fukkink, Minne Fekkes

**Affiliations:** 1 Research Institute of Child Development and Education, Faculty of Social and Behavioural Sciences, University of Amsterdam, Nieuwe Achtergracht 127, 1018 WS Amsterdam, The Netherlandsminne.fekkes@tno.nl (M.F.); 2 Center for Applied Research on Education, Faculty of Education, Amsterdam University of Applied Sciences, Wibautstraat 2-4, 1091 GM Amsterdam, The Netherlands; 3 TNO Child Health, Sylviusweg 71, 2333 BE Leiden, The Netherlands

**Keywords:** early childhood education and care (ECEC), process quality, socio-emotional development, longitudinal, meta-analysis

## Abstract

We investigated the relationship between process quality in early childhood education and care (ECEC) and children’s socio-emotional development in a meta-analysis of longitudinal studies. Our multi-level meta-analysis of 31 publications reporting on 16 longitudinal studies (N = 17,913 children, age: 2.5–18 yrs) demonstrates that the process quality of ECEC is a small but significant predictor of children’s socio-emotional development over time (ES = 0.103, SE = 0.026, *p* < 0.001, 95% CI: 0.052–0.155). This longitudinal association extends to the age of 18 years in our sample. Process quality of ECEC is, thus, a significant and stable predictor of children’s socio-emotional development and well-being from toddlerhood to adolescence. The longitudinal relationship was moderated by the type of care (center-based vs. home-based) and the informant (parent, professional caregiver, external assessor, or self-report of the child). Implications for future ECEC research are discussed.

## 1. Introduction

Research into the effect of early childhood education and care (ECEC) for preschool children on their socio-emotional development goes back several generations. The first generation of ECEC studies compared children who attended ECEC with children who stayed at home during the preschool period, and many of these studies did not include process quality measures [1,2]. Process quality refers to the interactions and experiences children have within a child care setting. This includes caregiver–child interactions, interactions with other children, and exploration of the physical environment, which support children’s well-being and stimulate their learning and development [3]. Specifically, sensitive interactions with emotional and instructional support from staff, positive peer interactions, and guided exploration during indoor and outdoor activities provide a rich and safe environment that stimulates children’s well-being and socio-emotional development.

Process quality is influenced by structural quality characteristics. Well-known structural quality characteristics include the caregiver-to-child ratio, group size, and caregiver education and training. These structural factors set the foundation for process quality, which has a direct impact on child development outcomes. Put differently, structural quality is a distal variable that influences process quality as a proximal variable, which directly influences child development. In a number of review studies, the importance of process quality has been demonstrated for children’s cognitive development [4,5] and other outcomes [6].

The first pioneering studies were followed by studies that included measures of the quality of ECEC, both structural quality characteristics (such as group size, staff–child ratio, and staff training) and process quality. The second generation of research showed that the process quality in particular predicted children’s development [7]. Fitting within a socio-ecological perspective, the third generation of ECEC research evaluated the effects of ECEC on children’s development, taking into account variables at the levels of the child, the family, and ECEC. The large-scale Study of Early Child Care and Youth Development (SECCYD) in the United States by the National Institute of Child Health and Human Development Early Child Care Research Network (NICHD ECCRN) represents a milestone in this last generation of longitudinal research into the effects of ECEC.

Different longitudinal studies into the effects of ECEC from the United States and other countries have been published [8], covering center-based care, home-based care, or both types of ECEC during the preschool period. These studies on the effects of ECEC have reported both positive and negative outcomes for the socio-emotional domain. Studies from the United States have found that frequent use of ECEC in the first year of life is a predictor of higher levels of problem behavior, negative moods, and aggression and conflict [9]. In the NICHD study, for example, more exposure to center-based care predicted more teacher-reported externalized problems, although the negative outcomes were modest. Parental assessments of the problem behaviors of their children did not show convergent findings [10,11]. Other studies, by contrast, have provided evidence of modest positive impacts of ECEC on children’s socio-emotional development. Some Scandinavian studies, for example, have reported positive effects for the socio-emotional domain. For example, three-year-old children who attended a child care setting played longer with peers and were more empathetic and prosocial toward other children [12,13]. Also, in a study on Australian ECEC, Gialamas and colleagues [14] reported a positive relationship between child care attendance and the emotional self-regulation of seven-year-old children.

### 1.1. Moderators of Longitudinal Effects of ECEC

In longitudinal studies, various researchers have explored whether the quality of child care may have a (stronger) effect on child outcomes for specific child populations. Studies from the diathesis–stress paradigm [15] have tested the interaction effects to explore whether some groups of children with specific traits (i.e., diatheses) may be more affected by the child care environment (i.e., ECEC as a stressor). In this line of study, researchers have not only investigated the main effects of ECEC quality but have also explored whether interaction effects (e.g., quality*SES, quality*gender, quality*race) predict variation in children’s socio-emotional outcomes. Studies from the differential susceptibility paradigm [15,16] have revealed that some phenotypic susceptibility variables [17] may moderate ECEC effects on socio-emotional outcomes. A study by Pluess and Belsky [18], which was the first to explore the differential susceptibility hypothesis in an ECEC context, demonstrated that the quality of ECEC was a significant predictor for children with a difficult temperament, whereas children with lower levels of negative affect were resilient to the quality of the child care environment. Specifically, children with high levels of negative affect had relatively few behavioral problems and good social skills in high-level ECEC, whereas peers with similar temperaments developed more behavioral problems and fewer social skills in low-quality ECEC [18,19]. In sum, studies from both the diathesis–stress paradigm and the differential susceptibility paradigm have revealed demographic and phenotypic susceptibility variables at the child level that significantly moderate the effect of quality on children’s development, including socio-emotional outcomes. These findings suggest that the association between ECEC quality and child outcomes is not necessarily a straightforward relationship (i.e., a simple main effect), but may involve a complex interplay of multiple variables that predict children’s outcomes.

In a small number of studies, researchers have also explored quality threshold effects in the context of ECEC. This quality threshold hypothesis, which has mostly been studied in the context of cognitive outcomes [20,21] assumes that high-quality ECEC may be necessary to improve outcomes at the child level, because mediocre or low-quality levels may not be sufficient to boost the development of young children. Thus, quality levels within study samples may moderate the relationship between process quality and socio-emotional outcomes, but between-study variation in (average) ECEC quality is also possibly related to developmental outcomes. The fact is that some studies have explicitly focused on the effects of high-quality ECEC [22], whereas other studies evaluated average low-to-medium quality levels of ECEC.

### 1.2. Review Studies into ECEC Effects on Children’s Development

Recently, some reviews with different focuses and designs have summarized the findings from primary studies into the effects of child care on children’s development. Reviews of longitudinal studies into the relationship between the quality of ECEC and cognitive outcomes have reported small but statistically significant effects. Perlman et al. [4], based on their meta-analysis of studies with CLASS, concluded that process quality is a small but significant predictor of children’s cognitive outcomes. Ulferts et al. [5] also reported a small but statistically significant relationship (*r* = 0.11) between the process quality of ECEC and children’s cognitive development, aggregating findings from European studies. Also, Von Suchodoletz et al. [6] found small associations between process quality indicators and most child outcomes, including a wide variety of outcome domains (e.g., social competence, behavioral problems, motor skills). These outcomes suggest, at least for the cognitive domain, a clear relationship between the quality of ECEC and children’s development. However, Howard et al. [23] recently argued that reviews and meta-analyses have reported conflicting findings regarding the assumed association between ECEC quality and children’s developmental progress. Based on their review, they concluded that current ECEC quality measures with different indicators are inconsistent predictors of child outcomes. Future meta-analyses are, therefore, important to estimate the reliability and magnitude of the association between quality dimensions and child-level outcomes, according to these authors, and this line of research should explore important moderators that may explain variation in the reported outcomes.

The inconsistency of findings may be related to different study characteristics. First, there may be variation in outcomes between countries because countries have different ECEC policies with different structural quality characteristics and varying levels of regulation [8,24]. Second, studies with effect sizes based on self-reported process quality measures have reported higher and more variable outcomes, compared to studies with observed process quality measures [6,25]. Different approaches to the measurement of process quality might have contributed to the inconsistent empirical evidence for the link between process quality indicators and child outcomes. Specifically, results from studies using self-reported quality measures may be inflated because self-perceived quality and some of the included child outcomes were reported by the same staff [6]. Third, Von Suchodoletz et al. [6] found some evidence of publication bias for the socio-emotional domain in their review, particularly for children’s behavioral and socio-emotional problems. Finally, ECEC effects have been investigated in a variety of study designs, and the reported outcomes vary between designs [23]. Some reviews have focused on the outcomes of quasi-experimental studies, often in the context of early education interventions [26,27], whereas other studies included non-experimental, longitudinal studies.

### 1.3. Goal of This Study

Although U.S. studies still predominate in the literature [6], several well-conducted longitudinal studies from the third generation have been published by researchers from other regions, including Australia and Europe. This state of affairs allows for a meta-analysis into the relationship between the process quality of ECEC and children’s socio-emotional development, aggregating data from longitudinal studies conducted in various countries to identify how the quality of ECEC affects children’s socio-emotional development throughout later childhood and into adolescence [9]. Our study tests the association between the process quality of child care for children between 0 and 4 years old (i.e., the independent variable) and children’s socio-emotional development in middle childhood and adolescence (5–18 yrs) (i.e., the outcome variable).

We explored, at the meta-analytic level, whether the relationship between ECEC quality and child outcomes is moderated by study characteristics. Specifically, we explored general study characteristics (U.S. study or other; NICHD or other), methodological characteristics (type of effect size, quality measure, outcome measure; informant), and child characteristics (age) (see also Table 1 and Table 2).

We also included the ECEC quality levels from individual studies to explore, at a meta-analytic level, whether the assumed relationship between ECEC and socio-emotional outcomes may be moderated by the quality level, as predicted by the threshold hypothesis (see Quality score, Table 2). Fitting in with the diathesis–stress paradigm and differential susceptibility hypothesis, we finally explored which variables may moderate the relationship between the quality of ECEC and children’s socio-emotional development by summarizing statistical interaction effects from the included primary studies (see Table 3).

## 2. Methods

We conducted a systematic literature search (undertaken in February 2018) of the PsycINFO, PubMed, SCOPUS, and Web of Science (SSCI) databases, using the PICO strategy [28] and PRISMA guidelines [29], to retrieve longitudinal studies published from 2000 onward concerning the relationship between the quality of ECEC during the preschool period (0–4 years) and children’s socio-emotional development.

**Figure 1:**
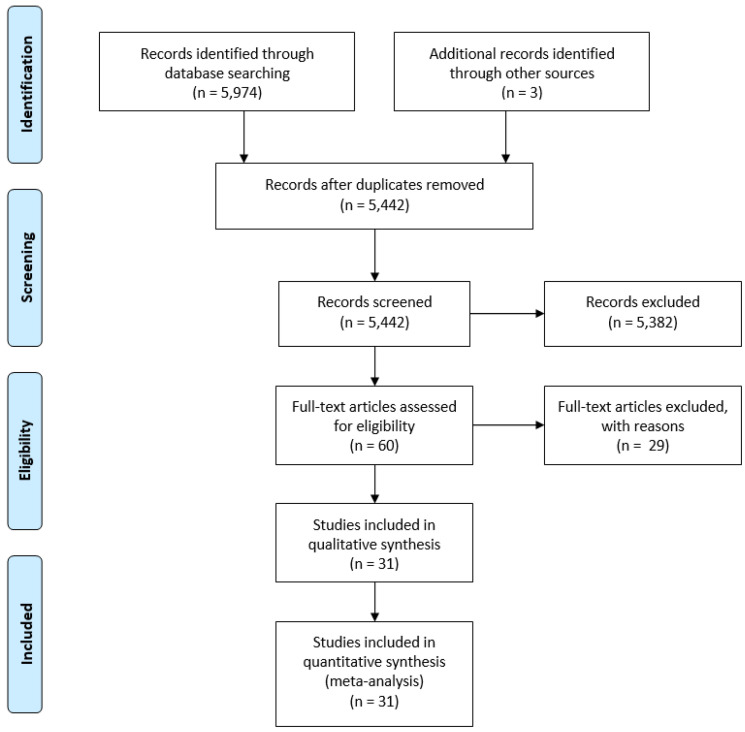
Search overview: PRISMA flow diagram.

The first author screened titles and abstracts. Titles and/or abstracts that raised questions were reviewed independently and then discussed by all three authors at fortnightly team meetings to validate eligibility choices. The three authors all read the full texts of the selected studies as a check of the final selection process [30]. The final sample included 31 studies from 16 different cohorts in which children were followed longitudinally (see Figure 1).

### 2.1. Coding

We coded the included studies for descriptive purposes (see Table 1). In addition, our coding of the sample and various methodological characteristics allowed for a moderator analysis to explore whether study outcomes were systematically related to these study characteristics (see Table 2). Each study was coded using an extensive scheme comprising general characteristics drawn from the meta-analytical literature (year of publication, country), methodological characteristics, and ECEC-specific variables. One of the ECEC-specific variables concerned the composition of the sample, i.e., whether the sample was composed of children attending center-based care, children attending home-based care, or a combination. We also coded whether the quality measure applied was the overall process quality (e.g., the Environmental Rating Scales, such as ECERS-R [31], ITERS-R [32] or the quality of staff–child interactions (such as CLASS, [33]) and ORCE, [15]).

For the quality measure, we coded the mean and the standard deviation; this was possible for 94 (73%) of the effect measures. The studies included in the final selection used different measurement tools with different maximum scores, so in order to compare their mean quality scores using a fixed metric, we expressed the mean score as a proportion of the maximum possible score (for example, an ECERS score of 4 with a maximum score of 7 is 4/7 = 0.57; similarly, an ORCE score of 2.5 on a scale with a maximum score of 4 is 2.5/4 = 0.63). Thus, this proportional measure provided a comparable overall indication of the quality level reported in each study, based on a common metric between 0 and 1. This score allowed us to explore, at a meta-analytic level, the possible threshold effect, e.g., is the longitudinal relationship between process quality and children’s outcomes stronger for higher-quality levels?

For the effect measure, the following items were coded: positive or negative outcome measure subdivided into (internalizing and/or externalizing) problem behavior or prosocial behavior; *r* or β (see also analyses below); and the informant (professional caregiver, parent, self-reporting, external observer, or some combination of these in one composite measure).

Each study was coded by two people (the first author plus the second or third author), and their independent assessments were then discussed item by item during multiple weekly sessions so as to arrive at an agreed final coding.

For our exploration of possibly differential effects of process quality on children’s development, we listed child-by-environment interactions from the primary studies (see 3.4). A number of child/family characteristics were important variables of the primary studies. Following Putnam et al. [34], temperament is defined as stable, biologically based individual differences in behavior that are relatively independent of children’s development. Some ECEC studies have focused on negative affect as a temperamental trait (also referred to as ‘difficult temperament’), which is a general tendency to experience negative emotions and is characterized by discomfort, fear or distress to novelty, anger/frustration, sadness, and low soothability. In a number of studies, risk scores at the child and/or family level were included in the analysis. These risk scores were based on well-known but heterogeneous sets of risk factors for children’s development in their early years (e.g., low socio-economic status, living in a poor neighborhood, single-parent family); we refer to the individual studies for the exact definition of ‘risk’.

### 2.2. Analyses

Taking into account the hierarchical structure of the data [35], with individual effect measures for different socio-emotional outcomes (level 1) nested within a particular wave (level 2) of a sample tracked over a longer period of time (level 3), results/data were analyzed using a multilevel model [36,37]. The effect sizes—coming from studies with different samples and research designs—were analyzed using a random effects model, which assumes that the observed effect sizes may vary across studies due to differences in the investigated correlation as well as sampling variability [38]. The analyses were performed using the Metafor package for R [39], with the help of a tutorial by Assink and Wibbelink [40]. All the models were estimated using the restricted maximum likelihood method, which is the recommended method for estimating the heterogeneity of variance [39].

The effect measure used in our meta-analysis was Pearson’s correlation r, which indicates (again, in our meta-analysis) the relationship between process quality and children’s socio-emotional development. In the included studies, process quality was always reported as a “positive” variable. However, the socio-emotional outcome measures could be either a “positive” outcome (k = 60, 46.9%; e.g., prosocial behavior) or a “negative” one (k = 68, 53.1%; e.g., internalizing and externalizing problem behavior). Statistics for negative child outcomes were consistently converted by changing the sign. The aggregate effect measure from this meta-analysis reflects the positive relationship between the quality of ECEC and children’s favorable socio-emotional development.

The effect measure *r* was taken from the research report wherever possible, and in other cases, the standardized beta coefficient was converted to *r*. The correlation coefficient *r* and the beta coefficients from multiple regression models were found to be strongly correlated (*r* = 0.84 in the review study by Peterson & Brown [41]). In this meta-analysis, too, the correlation between beta coefficients and converted *r* values was found to be very strong (*r* = 0.944, *p* < 0.001), with comparable mean sizes and deviation (M*r* = 0.074, SD*r* = 0.097, k = 88; Mβ-to-*r* = 0.103, SDβ-to-*r* = 0.206, k = 40, F(1, 126) = 1.145, *p* = 0.287). The statistical parameters were taken from the most complete regression models in each study (often referred to as the ‘final model’ in the original reports). For a small number of studies, alternative effect sizes from the reports (e.g., odds ratio for dichotomous variables) were converted to *r*.

Following the recommendation made by Bowman [42], *r* values were converted to Fisher’s z for the aggregation of all outcomes. This transformed variable follows a normal distribution and allows for the calculation of a confidence interval. Finally, this z value was then converted back to *r* for interpretation of the outcomes. The Fisher transformations resulted in hardly any changes in our study, as the majority of the values were relatively small; in only two cases could an outlier with a value > 0.50 not be converted to z; these two extreme values (1.6%) were adjusted to the maximum value of 0.50 through winsorizing.

The heterogeneity of the outcomes was tested using Q and I^2^ [43]. Following the guidelines of Cooper [44], moderators were tested individually during the moderator analysis, taking into account possible multicollinearity and aiming to maintain statistical power. In the analyses, we tested whether the coded methodological characteristics and our choices (such as converting beta to *r* and the distinction between “positive” and “negative” outcome measures) moderated the relationship between process quality and children’s socio-emotional development. The F test was used to determine the significance of moderators, with the residual variance QE test applied in each case.

Finally, the validity and generalization of conclusions may be affected by publication bias (i.e., if the chance of a study being published by a scientific journal is associated with the statistical significance of findings or discontinuation of a longitudinal study after non-significant results). In a sensitivity analysis, we tested for publication bias with the visual inspection of a funnel plot, which presents effect sizes plotted against their standard errors and the commonly used Egger’s test based on this plot [45]; in case of publication bias, there is a funnel plot asymmetry and a significant Egger’s test.

## 3. Results

The sample consisted of longitudinal data from 31 studies, conducted in eight countries (Australia, Canada, the Netherlands, France, Norway and Sweden, the United Kingdom, and the USA), with children who attended center-based, home-based, or ‘non-parental’ care (see Table 1 for an overview).

**Table 1:** Study characteristics and moderators for the included studies.

Authors (Year of Publication)	Country	Sample Size *	Cohort Name	Type of Care	Age of Children (Months) *	Outcome Category	Informant	Type of Quality	Proportion Quality Score	No. of *ESs*
[15]	USA	1073	NICHD	Non-parental care	144	Social development Externalizing problem behavior	Staff	Process–overall	0.73	4
[18,19]	USA	842	NICHD	Daycare	180	Externalizing problem behavior	Self-report	Process–overall	0.71	3
[46]	USA	828	Family Life Project (FLP)	Non-parental care	60	Social development	Staff	Process–sensitive responsivity/positive interactions	-	1
[47]	Netherlands	230	Pre-Cool	Daycare	36	Externalizing problem behavior Social development	Staff	Process–overall	0.72	2
[48]	USA	1175	Family Life Project (FLP)	Non-parental care	78	Problem behaviorSocial development	Staff	Process–overall	0.71	5
[49]	Netherlands	180	Pre-Cool	Daycare	41	Externalizing problem behaviorSocial developmentInternalizing problem behavior	StaffParent	Process–overall	0.76	4
[50]	USA	957	NICHD	Non-parental care	180	Externalizing problem behavior	Self-report	Process–overall	0.73	3
[51]	Sweden	52	Goteborg Child Care Study	Non-parental care	180	Social development	Composite score of multiple informants Staff Self-report	Process–overall	-	3
[52]	USA	146	NICHD	Non-parental care	36	Problem behavior Social development	StaffParent	Process–overall	0.71	4
[14]	Australia	1038	Longitudinal Study of Australian Children	Non-parental care	82	Social development	Parent	Other	0.93	2
[53]	Australia	1282	Longitudinal Study of Australian Children	Non-parental care	30	Problem behavior	ParentStaff	Other	0.93	2
[54]	USA	107	Not specified	Family daycare	51	Internalizing problem behavior	Composite score of multiple informants Observation	Process–overall	-	2
[55]	UK	2862	Effective provision of preschool education project (EPPE)	Daycare	58	Social development	Staff	Process–overall	-	2
[56]	USA	851	Educare Implementation Study (ELN)	Daycare	36	Social developmentProblem behavior	Staff	Environmental rating scales	0.80	4
[57]	USA	1364	NICHD	Daycare	54	Social development	Observation	Process–overall	-	8
[58]	USA	1400	Early Childhood Longitudinal Study (ECLS)	Non-parental care	72	Social development Externalizing problem behavior	Staff	Environmental rating scales	0.60	2
[59]	Canada	70	Young Children and Their Living Environments Canada	Non-parental care	48	Internalizing problem behavior Externalizing problem behavior	Parent	Environmental rating scales	0.67	2
[60]	USA	451	TANF (Temporary Assistance for Needy Families)	Non-parental care	48	Social development	Parent	Process–sensitive responsivity/positive interactions	0.69	1
[61]	Norway	881	Better Provision for Norway’s Children (BePro)	Daycare	63	Social development	Staff	Environmental Rating Scales	0.69	6
[62]	USA	1201	NICHD	Non-parental care	36	Social development Externalizing problem behavior	Parent Staff Observation	Process–overall	-	15
[63,64]	USA	794	NICHD	Non-parental care	36	Social development Problem behavior	ParentStaff	Process–overall	0.71	4
[65]	USA	1058	NICHD	Non-parental care	54	Social development Externalizing problem behavior	ParentStaff	Process–overall	0.70	7
[66]	USA	1095	NICHD	Non-parental care	54	Social development Problem behavior	Parent Staff Observation	Process–overall	0.73	10
[67]	USA	975	NICHD	Non-parental care	102	Social development Externalizing problem behavior	Self-report Parent Observation Staff	Process–overall	0.72	14
[68]	Switzerland	89	Lausanne CaMie and OLiVE Study	Non-parental care	36	Social development Problem behavior	Observation Parent	Process–overall	0.59	3
[69]	UK	996	Families. Children and Child Care (FCCC) Study	Non-parental care	51	Problem behavior	Staff Parent	Process–overall	-	2
[11]	USA	958	NICHD	Non-parental care	180	Externalizing problem behavior	Self-report	Process–overall	0.73	3
[70]	USA	779	NICHD	Non-parental care	221	Externalizing problem behavior	Self-report	Process–overall	0.73	3
[71]	USA	204	Three-City Study	Non-parental care	40	Problem behavior Social development	Parent	Process–overall	0.68	2
[72]	USA	349	Three-City Study	Non-parental care	111	Internalizing problem behavior Externalizing problem behavior	Parent	Process–overall	0.78	2
[22]	USA	5037	Educare Implementation Study (ELN)	Daycare	49	Social development Problem behavior	Staff	Environmental rating scales	0.79	3
Summary scores	USA: 68% Other: 32%	*M*: 946 (*SD* = 580)		Non-parental care: 22 (71%) Daycare: 8 (26%) Family daycare: 1 (3%)	*M*: 79 (*SD* = 55) *Min*–*max*: 30–221	Internalizing: 4× Externalizing: 13× Problem behavior: 10× Social development: 22×	Composite: 2× Observation: 6× Parent: 15× Self-report: 6× Staff: 19×	Process overall: 71% Process sensitivity: 6% ERS: 16% Other: 6%	*M*: 0.73 (*SD* = 0.08). *Min*–*max*: 0.59–0.93	*M*: 4.1(*SD* = 3.4) *Min*–*max*: 1–15

Note. *: The sample size and age of the children refer to the last wave of the longitudinal studies.

In total, 17,913 children were included in the sample with measures of socio-emotional development at different ages, varying from 2.5 to 18.4 years, with an average of 6.6 years (*SD* = 4.6). A variety of measures were used in the studies, including problem behavior (internalizing and externalizing) and prosocial behavior. The measures were often related to classroom misconduct, aggression, or antisocial behavior as negative outcomes, or pro-social behavior and peer relationships as positive outcomes. Most socio-emotional outcomes shared a focus on social behavior in both formal and informal environments with peers and/or adults. The informant was often the parent or professional staff from either ECEC or the school. Some studies included (additional) alternative measures (e.g., observation, a composite score).

The quality score was, on average, 0.73 (*SD* = 0.09) on a scale from 0 to 1, varying from 0.59 to 0.93. Put differently, the quality of the ECEC was, on average, three-quarters of the maximum level achievable. This corresponds roughly with a score just above 5 on the original seven-point scale of the ERS and CLASS measures (i.e., 7 * 0.73, corresponding to a ‘mid-range’ score for CLASS and ‘good quality’ for ERS, like the ITERS and ECERS) and a score just below 3 for the four-point scale of the ORCE measure (i.e., 4 * 0.73, corresponding to ‘moderately low’ for the ORCE).

### 3.1. Overall Effects and Heterogeneity

The aggregate effect size *r* from our meta-analysis is 0.103 (SE = 0.026, *t* = 3.95, *p* < 0.001, 95% CI: 0.052–0.155), indicating a small but statistically significant effect of process quality on the socio-emotional development of children attending ECEC. The variance at the effect measure, measurement, and sample level were 0.010, 0.009, and 0.002, respectively (with corresponding sample sizes of 128 measures, 39 waves of measurement, and 15 samples). The outcomes of the studies were not homogeneous; Cochran’s *Q* (*df* = 127) = 2288.2, *p* < 0.001, *I*^2^ index = 94.4%.

### 3.2. Moderator Analysis

With a meta-regression analysis, we investigated which variables were associated with the effect sizes and might explain some of the heterogeneity in study outcomes. The longitudinal relationship found between quality and children’s socio-emotional development was moderated by two out of the investigated variables (see Table 2). In addition, there was one trend effect (defined as a finding with 0.05 < *p* < 0.10).

**Table 2:** Outcomes of moderator analysis.

Moderator	*r* for Subgroup	Significance	Δ*Q*	*Q*E
Study from the U.S.; other	0.113 0.094	*F*1, 126 = 0.137 *p* = 0.712	35.2	2253.0 ***
NICHD study; other	0.078 0.111	*F*1, 126 = 0.194 *p* = 0.661	11.0	2277.2 ***
Effect size *r* from report; effect size *r* converted	0.094 0.114	*F*1, 126 = 0.226 *p* = 0.663	14.7	2273.5 ***
Quality measure: teacher–child interaction Quality measure: global process quality	0.124 0.027	*F*1, 126 = 2.257 *p* = 0.136	42.0	2246.2 ***
Sample: center-based care only; sample: home-based care only; sample: both	−0.002 0.240 0.143	*F*2, 125 = 7.102 ** p = 0.001	370.1	1918.1 ***
Outcome measure: problem behavior Outcome measure: prosocial behavior	0.118 0.092	*F*1, 126 = 1.180 *p* = 0.279	104.7	2183.5 ***
Informant: professional; informant: parent Informant: external observation; informant: self Informant: multiple	0.117 0.111 −0.017 0.055 0.145	*F*4, 123 = 4.080 ** p = 0.004	413.6	1874.6 ***
Age of child at wave of data collection (centered)	−0.000/yr	*F*1, 126 = 0.028 *p* = 0.867	9.2	2279.0 ***
Quality score (proportion of maximum score)	0.561	*F*1, 93 = 3.267 *p* = 0.074	-	725.3 ***

Note: Year of publication is, on average, 2009 (*SD* = 6.0); age of child is, on average, 70.9 months (*SD* = 48.9), *min–max*: 24–220); **, ***: *p* is < 0.05, < 0.01, or < 0.001, respectively.

First, the relationship appeared stronger when home-based care was included in the study, although it should be noted there was one study with only home-based care (*r* = 0.240, 2 effect sizes, 1.6%), as opposed to center-based care only (*r* = −0.002, 25.8% of the effect sizes). The largest part of the studies included a mixed sample with home-based and center-based care, described as non-parental care in Table 2 (*r* = 0.143, 72.7%).

Secondly, the longitudinal relationship between quality and socio-emotional development appeared stronger with adult-reported measures (i.e., a parent or a professional caregiver, 25.8% and 44.5% of the effect measures, respectively), compared to self-reports (11.7%) or measures with an external assessor who observed the child during a visit (16.4%).

**Figure 2:**
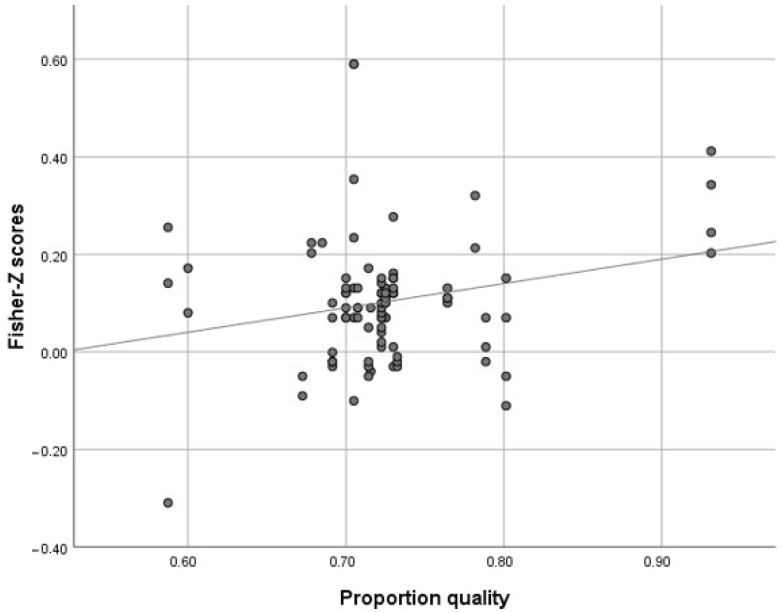
Scatter diagram of correlation coefficient (with Fisher’s *r*-to-*z* transformation) for the longitudinal relationship between quality of ECEC and children’s socio-emotional development, set against the quality level (proportional score, *min–max*: 0–1).

The third moderator approached statistical significance and is, therefore, only a trend effect (*p* = 0.074). For a subset of 95 effect sizes (i.e., 74.2% of the total sample of 128), we could determine a quality score expressed as a proportion of the maximum possible score. This quality score showed, as expected, a positive relationship with socio-emotional outcomes (see Figure 2).

The relationship between process quality from ECEC and children’s socio-emotional development did not change as children grew older (*p* = 0.867). Hence, child age, which varied from 24 to 221 months in our sample, did not moderate the strength of the longitudinal association between ECEC quality and socio-emotional outcomes. The effect sizes from our review were not related to the methodological choice of determining effect sizes based on either the correlation coefficient (68.8%) or regression weights (31.3%). Moreover, the converted effect sizes for “negative” outcome measures (i.e., problem behavior, 43%) were not significantly larger than those for positive outcome measures (*r* = 0.118 and 0.092, respectively, with a non-significant difference, *p* = 0.279). Finally, the relationship between the year of publication and the strength of the relationship (−0.009/year, *F*1, 126 = 7.033, *p* = 0.009, Δ*Q* = 98.3, *QE =* 2189.9) appears to be caused by only two negative outliers (see Supplementary Figure S1).

### 3.3. Sensitivity Analysis

**Figure 3:**
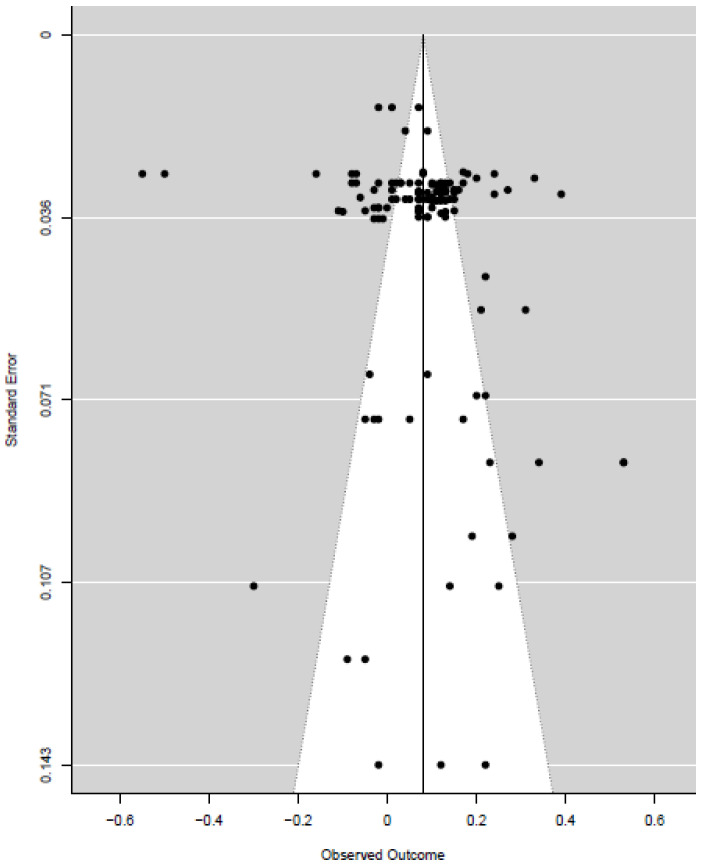
Funnel plot of the effect sizes against their standard error.

The effect found in our study did not appear to be strongly influenced by publication bias, according to the Egger regression, *t* = 1.90, *df* = 126, *p* = 0.060. The funnel plot (Figure 3) showed no clear asymmetry in the distribution of effect sizes around the mean effect (indicated by the vertical line). Also, the outcomes outside the 95% confidence interval (indicated by the white field) were distributed more or less equally to the left and the right of the vertical line.

The moderator analysis further revealed that the aggregate results of our meta-analysis did not appear to be influenced by the NICHD study, which was dominant in our dataset (60.9% of the results), or by U.S. studies in general in our sample.

### 3.4. Differential Effects: Child-by-Environment Interactions

A number of studies from our sample investigated whether ECEC quality may have differential effects on the socio-emotional development of specific subgroups of children (e.g., boys vs. girls, children with a difficult vs. easy temperament). This subset of studies analyzed whether the effect of quality of ECEC on socio-emotional outcomes was moderated by variables at the child, parent, family, or child care level by testing whether an interaction effect of quality of the ECEC with other variables was statistically significant. Table 3 presents an overview of 104 interaction effects (both significant and non-significant) from the included studies, categorized by susceptibility factors at the child, parent, family, or ECEC level.

**Table 3:** Overview of significant child-by-environment interactions from the included longitudinal studies.

Study	Child/Environmental Factor	Measure	Outcome	Informant	Effect Size	se	Effect of Quality on Outcome for Levels of Moderator
[70,73]	Child: difficult temperament	ORCE	Externalizing problem behavior	Self-report	−0.070 ^a,^*	-	Significant relationship between quality and externalizing problem behavior for difficult temperament (β = −0.17) vs. no difficult temperament (β = −0.02).
[48]	Child: affective self-regulation	CLASS	Social skill	Teacher	−0.160 ^b,^*	0.07	Significant relationship between quality and social skills for low- (B = 0.25) vs. high-affective self-regulation (B = 0.09).
	Child: gender	CLASS	Social skill	Teacher	−0.200 ^b,^***	0.06	Significant relationship between the quality and social skills for boys (B = 0.17) vs. girls (B = −0.03).
[50]	Quantity of care	CLASS	Externalizing behavior	Parent	−0.060 ^b,^**	0.02	Significant relationship between quality and externalizing behavior for low vs. high amounts of care.
[54]	Income	LSAC	Behavioral difficulties	Parent	-	-	No relationship between quality and behavioral difficulties for low vs. high income.
	Income	LSAC	Behavioral difficulties	Teacher	-	-	Significant relationship between quality and behavioral difficulties for low vs. high income.
[56]	Child: developmental risk factors	CIS	Self-regulation	Teacher	0.040 ^b,^**	-	Experiencing higher-quality preschool for a longer duration predicted positive effects on children’s self-regulation.
	Child: developmental risk factors	CIS	Antisocial behavior	Teacher	−0.060 ^b,^***	-	Experiencing higher-quality preschool environments for a longer duration predicted positive effects on children’s antisocial behavior
[65]	Sociocultural risk mother	ORCE	Prosocial behavior	Parent	0.020 ^b,^*	0.01	Mother’s prosocial behavioral ratings were not related to sociocultural risk when child care quality was high but were negatively related to sociocultural risk when quality was low.
[67]	Quality of parenting	ORCE	28 outcome variables	Parent/teacher/observer	-	-	Seven significant interaction effects across 28 outcomes and 84 interaction tests, involving child care quality, quantity, and parenting quality. No consistent pattern emerged to suggest that child care was associated with more optimal outcomes for the lowest parenting quartile vs. the highest parenting quartile.
[18]	Child: negative emotionality	ORCE	Behavior problems in pre-K	Teacher	−1.410 ^b,^**	-	Significant relationship between quality and behavioral problems for low (β = −0.01) vs. high negative emotionality (β = −0.26).
	Child: negative emotionality	ORCE	Social skills pre-K	Teacher	1.020 ^b,^*	-	Significant relationship between quality and social skills for low (β = 0.03) vs. high negative emotionality (β = 0.14).
[19]	Child: negative emotionality	ORCE	Teacher–child conflict	Teacher	−2.610 ^b,^*	-	Significant relationship between quality a teacher–child conflict for low (β = −0.004) vs. high negative emotionality (β = −0.25).
	Child: negative emotionality	ORCE	Behavior problems	Teacher	−3.870 ^b,^*	-	Significant relationship between quality and behavior problems for low (β = −0.04) vs. high negative emotionality (β = −0.33).
[71]	Quantity of care	CIS and ECERS	Problem behavior	Parent	−0.150 ^b,^*	0.06	An increase in the number of hours children spent in care was associated with reductions in behavioral problems among children in high-quality (vs. low-quality) child care arrangements.
[72]	Child: gender	ECERS	Internalizing behavior	Parent	−3.050 ^b,^*	1.43	High-quality child care appeared to be especially protective for boys’ (vs. girls’) development of internalizing behavior problems.
	Child: Afro-American	ECERS	Externalizing behavior	Parent	−3.130 ^b,^*	1.51	Child care quality was especially protective against the development of behavioral problems among African American (vs. Hispanic) children.

Note. ^a^ = standardized coefficient from report; ^b^ = unstandardized coefficient from report; * *p* < 0.05, ** *p* < 0.01, *** *p* < 0.001.

In our review of interaction effects, we found only modest support for the differential effects of ECEC quality on children’s socio-emotional development. The majority of the 104 statistical interaction effects summarized in our overview were non-significant (86%), and only 17 of the interaction effects (14%) were statistically significant. Most significant interaction effects involved susceptibility factors at the child level, including temperament (6 interaction effects), gender (2), child risk score (2), and race (1). The other reported significant interaction effects (*k* = 6) were related to low family income (2), quantity of child care (2), parenting (1), and a risk score at the family level (1). The quality measures from these significant interaction effects mostly pertained to the interaction skills of the caregivers. Furthermore, the significant interaction effects mostly involved externalizing problem behavior, including teacher- and parent-reported behaviors. No differential effects of ECEC quality were found related to children’s age, parenting (both sensitivity and cognitive stimulation), education of the parent, bilingual families, risk factors at the family level, continuity of care, type of care (i.e., home-based or center-based), and number of care arrangements. To conclude, the strongest pattern that emerged from our analysis of possible differential effects of child care quality on children’s socio-emotional development is that the combination of children with a difficult temperament as a susceptibility factor, with a quality measure focusing on teacher–child interaction as an environmental factor, was related to the children’s externalizing problem behavior as perceived by a parent or teacher. Specifically, the significant interaction effects indicated that higher-quality ECEC is more beneficial for children with a difficult temperament and children with a higher risk score. Two significant interaction effects were related to the quantity of child care, namely, an increase in child care hours was associated with reductions in children’s behavioral problems for children in high-quality child care.

As mentioned before, the large majority of explored interaction effects from the primary studies (i.e., 86%) was not significant. It should be noted that the abovementioned variables from the significant interaction effects were also involved in non-significant interaction effects. For example, the interaction between children’s negative affect and ECEC quality was also represented among the non-significant effects. Relatedly, some studies found that negative affect as a phenotypic susceptibility factor did not moderate the outcomes for teacher-reported externalizing problem behavior. In fact, this divergent pattern of both significant and non-significant outcomes was also visible within an individual study (see, for example, studies reporting on the NICHD study). As Table 3 shows, the strongest support for moderating effects was found for child risk scores (2-to-0 ratio for significant vs. non-significant interaction effects, respectively), temperament (6-to-5 ratio), and child care quantity (2-to-3). The ratio of significant vs. non-significant interaction effects was more skewed for gender (2-to-7), low income (2-to-7), race (1-to-6), parenting (1-to-11), and family risk (1-to-14). No significant interaction effects were reported in the literature for the other variables from Table 3 (age of the child, cognitive stimulation in the family, bilingual families, education of the parent, continuity of care, type of care, and multiple care arrangements).

## 4. Discussion

Longitudinal research shows that the quality of ECEC makes a positive contribution toward children’s socio-emotional development from toddlerhood to adolescence, up to the age of 18. The aggregated effect size from our meta-analytic review is around 0.10, which corresponds to a small effect according to Cohen’s [74] rule of thumb. The longitudinal relationship between ECEC quality and children’s socio-emotional development was found in several studies and across different operationalizations of both process quality and child outcomes from American, Australian, and European studies (British, Dutch, French, Norwegian, and Swedish) with different child care systems published between 2001 and 2018. The findings from the NICHD study are dominant in our sample, but various other studies have reported corresponding findings, and together they provide broad empirical support for the positive longitudinal relationship between ECEC quality and children’s socio-emotional development. This overall result also implies that, despite variation among individual studies [6,23], the current quality measures in the ECEC field show predictive validity at the meta-analytic level. This overall finding for the socio-emotional domain fits in with other reviews that supported the assumed link between ECEC quality and the cognitive domain [4,5,6].

It should be noted that our review is limited to the first two decades of the 21st century, i.e., a period that is characterized by the rapid growth of ECEC worldwide and the publication of the first longitudinal studies into the effects of child care on child development from different countries. Our review summarizes the findings for this historical period and provides a baseline for future research.

During this period, ECEC was a rapidly expanding (but still selective) service in many countries. In more recent ECEC policies, there have been global investments aimed at improving quality [3] and an international trend toward the integration of ECEC systems with education [75]. This has resulted in expanding enrollment rates for young children and near-universal ECEC for toddlers from various socio-economic backgrounds in a growing number of OECD countries [3]; this trend is intensified by the decreasing size of cohorts of children due to demographic decline in some countries [76]. The relatively recent but profound transformations in various nations (approximately taking place in the period 2005–2015, according to [76]) mark a new phase of ECEC in this decade. In fact, the growing public provision of ECEC, with relatively stable quality levels across OECD countries [76], also means a shift in scientific research. More universal ECEC with higher access for children with different backgrounds may result in a stronger, positive relationship between process quality and child outcomes because children from socio-economically disadvantaged households show greater gains from participating in universal, high-quality ECEC than their advantaged peers [77]. If ECEC quality levels may not only be stable but even gradually increase in the next years, this may also strengthen the relationship between process quality and children’s outcomes (see also the threshold hypothesis below).

Our meta-analysis demonstrates the assumed positive relationship between the process quality of ECEC and children’s socio-emotional development. This positive relationship extends to the current ECEC. This means that investing in the process quality of current ECEC contributes not only to the well-being of toddlers but also to that of a new generation of youth. Possibly, future studies may find stronger and more robust associations for a wider population, which would further highlight the importance of high-quality ECEC.

In our moderator analysis, we did not find a “fade out” pattern of effect sizes at later ages, and the positive association between the quality of ECEC and socio-emotional development seems to continue into early adulthood. Seen from this perspective, the quality of ECEC has a modest but sustainable effect on socio-emotional development. High-quality child care seems to contribute toward positive social development with more prosocial behavior, and less internalizing and externalizing problem behavior, acknowledging the modest association. Seen from this perspective, the quality of ECEC during children’s early years casts its shadow far into the future of children (see also [77]).

The moderator analysis provided only partial support for a dose–response relationship, as predicted by the threshold hypothesis [20,21]. The trend effect from our review tentatively suggests that the positive relationship between quality and socio-emotional development is stronger at higher-quality levels. This pattern is in line with the threshold hypothesis. However, a broader sample of studies with varying quality levels is needed to support this hypothesis more convincingly at the meta-analytic level. In addition, our findings support a positive linear relationship between child care quality and longitudinal outcomes in the socio-emotional domain, but it is not possible to specify a specific threshold level for the ‘average’ measure (i.e., expressed as a proportion score).

Our review further suggests that process quality from home-based care is more strongly related to the socio-emotional development of youth than center-based care. One possible explanation is that the “structure–process–outcome” relationship [78] may be moderated by the type of child care, with structural characteristics of home-based care—like a smaller group size and a more favorable caregiver-to-child ratio—potentially increasing the direct influence of process quality on children’s development. An alternative explanation is that current measures may more accurately capture the process quality that children receive in smaller home-based settings than in center-based settings. The stability of the individual caregiver in home-based care (compared to multiple and occasionally changing caregivers in center-based care) and the relatively small group of children (compared with the larger groups in center-based care) may contribute to a relatively accurate evaluation of quality in this child care setting. Future comparative studies should shed light on these tentative explanations and the possibly greater role of process quality of home-based versus center-based care for children’s socio-emotional development.

Finally, our review also included a summary of various statistical interaction effects that different researchers have explored to find out whether quality may have differential effects for specific subgroups of children. We found that only a small portion of the reported interaction effects was statistically significant (i.e., 16%), despite the large sample size from a number of studies. We found some evidence that if the quality of the caregiver–child interactions in ECEC is at a lower level, children with a difficult (vs. “easy”) temperament may develop more problem behavior during youth, as reported by parents or teachers. However, our review of statistical interaction effects with the process quality of child care does not provide comprehensive support for a specific “risk” factor (from a diathesis–stress perspective) or a “plasticity” factor (from a differential susceptibility perspective) at the child level that is consistently linked to socio-emotional outcomes from longitudinal ECEC research. Based on this review, it seems safe to conclude that the quality of ECEC generally has a similar (i.e., small but positive) effect on the socio-emotional outcomes for a broad population of youth, although there is limited evidence of differential, positive effects of high-level quality ECEC for children with “difficult” temperament, boys, and children from lower-income families.

### Limitations and Suggestions for Future Research

Our review demonstrates the significant relationship between the process quality of early-year child care and children’s socio-emotional outcomes from a longitudinal perspective. The changing ECEC context with the emergence of stronger, more integrated systems for a broader child population requires new empirical research into the relationship between process quality and child development. This line of research may profit from new meta-analytic reviews as well. Specifically, individual participant data meta-analysis may be helpful in demonstrating moderation effects in the context of a wider ECEC population with children from different socio-economic backgrounds. In this line of research, ECEC contexts and children’s developmental outcomes can be studied at the individual child level (see [79], for example), and this analytic approach is more precise than exploring moderators at the study level with meta-regression, as we used in our review (e.g., exploring home- vs. center-based care). This new approach can be complemented in international reviews with statistics on the availability, accessibility, and affordability of ECEC [80], as well as other ECEC system characteristics, like ECEC system integration [81]. This line of meta-analytic research with individual data at the child level and mapping of the increasingly available data on national ECEC systems in a comparative approach [75,76] allows for new ways of investigating child care effects in future reviews.

Whilst the empirical knowledge base has expanded, the longitudinal NICHD study remains dominant in the current literature as the source of multiple publications. Future meta-analytic research should, therefore, add new studies to the literature in order to gain a broader picture of the generalizability of the reported findings.

The effect sizes from our meta-analysis were derived from studies using regression models with different predictors. A common denominator of these models is that they included covariates at the child, parent, and family levels, but the models were not identical in terms of the theoretical constructs or their operationalization; we used a random model to account for this variation.

The statistical power of our moderator analysis is limited. Our moderator analysis suggests that the contribution of ECEC quality to children’s development may be stronger for home-based care than center-based care, but more research is needed to draw a clear conclusion. A complicating factor in our meta-analytical review is that home-based care has been studied less often, and some of the included studies aggregated findings for home- and center-based populations into ‘non-parental’ or ‘non-maternal care’. Future studies with a direct comparison between children’s development in home-based versus center-based care are needed to gain a better understanding of the differential effects of these two types of ECEC in the early years. The limitations from our analysis also apply to the non-significant “fading out” of effects at a later age, which may be heavily influenced by the small number of studies that followed children during a longer time frame. The low statistical power of our moderator analysis complicates the interpretation of our findings.

Finally, the observational measures from the included studies vary in the extent to which they apply to individual children and the amount of children’s daily experiences they sample. The measurement error in the quality measures may weaken the relationship between process quality and child outcomes. Also, other studies have reported modest predictive validity of process quality measures, like CLASS [4], ECERS [82], and various self-evaluation measures in child care [83].

## 5. Conclusions

This meta-analysis into the effects of process quality for the socio-emotional domain underlines the importance of high-quality caregiver–child interactions for children’s well-being and socio-emotional development for children and adolescents. Our findings fit with the outcomes of other recent reviews on longitudinal studies for the cognitive domain. Recent research highlights, at a meta-analytic level, the vital importance of process quality for the broad development of children in early childhood education and care from the early years to adolescence.

Investing in the process quality of ECEC matters not only for the well-being of young children but also for the socio-emotional development of children during middle childhood and adolescence. Meta-analytic reviews have demonstrated that high-quality in-service programs have the ability to improve the pedagogical quality and teacher–child interactions in the domain of emotional support [84], and this quality improvement is a key mechanism to accelerate the development of young children [85]. Both pre-service and in-service professional development of staff seem to be promising ways to improve process quality and stimulate children’s socio-emotional development.

## Data Availability

No new data were created or analyzed in this study.

## Supplementary Materials

The following supporting information can be downloaded at https://www.mdpi.com/article/10.3390/ijerph22050775/s1, Figure S1: Scatter Diagram of the Correlation Coefficient (with Fisher’s *r*-to-*z* Transformation) for the Longitudinal Relation between the Quality of Early Childcare and Children’s Socio- emotional Development, Set against Year of Publication.
