# Supplementary material for: The Impact of Process Quality in Early Childhood Education and Care on Socio-Emotional Development: A Meta-Analysis of Longitudinal Studies
Source: Int J Environ Res Public Health. 2025 May 14;22(5):775. doi: 10.3390/ijerph22050775 (PMC12111270; doi:10.3390/ijerph22050775)
Supplement: Supplementary file 1 [file ijerph-22-00775-s001.zip › ijerph-3532305-supplementary.pdf]

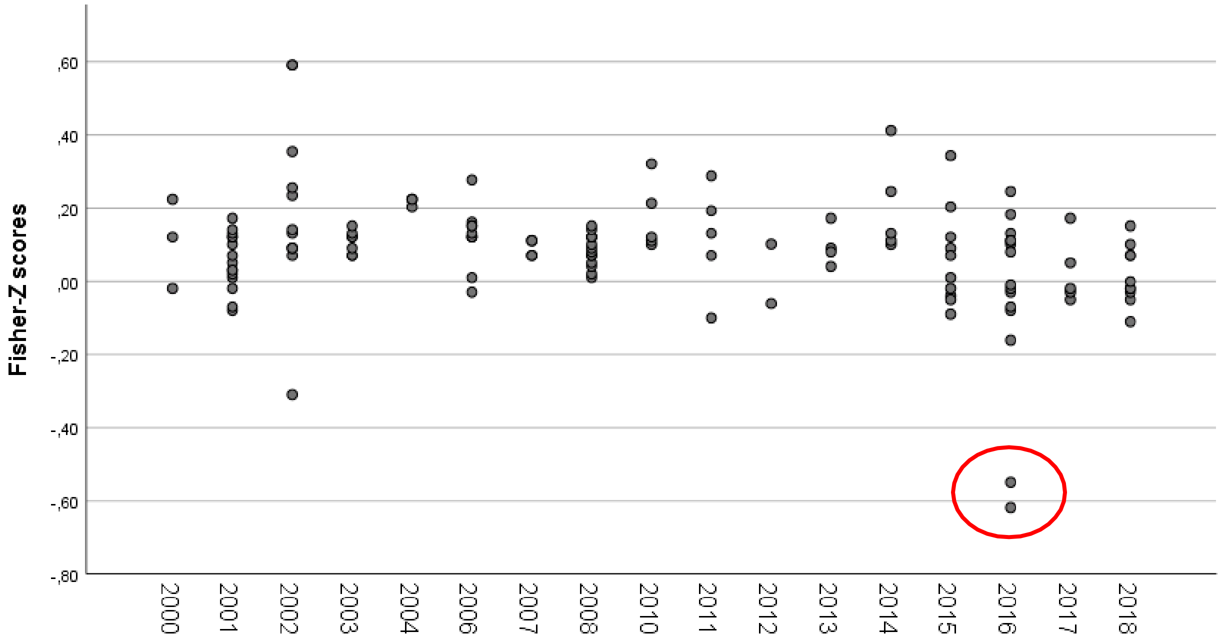

Figure S1. Scatter Diagram of the Correlation Coefficient (with Fisher's *r*-to-*z* Transformation) for the Longitudinal Relation between the Quality of Early Childcare and Children's Socio-emotional Development, Set against Year of Publication.
